# Siblings of FBXL4-related mitochondrial DNA depletion syndrome, leading to fatal fulminant pneumonia

**DOI:** 10.1016/j.ymgmr.2025.101266

**Published:** 2025-10-10

**Authors:** Takato Akiba, Shino Shimada, Shimpei Matsuda, Shoji Ishida, Yosuke Baba, Atsushi Yamashita, Hiromichi Shoji, Yasushi Okazaki, Kei Murayama

**Affiliations:** a Department of Pediatrics and Adolescent Medicine, Graduate School of Medicine, Juntendo University, Tokyo, Japan; b Department of Clinical Genetics, Graduate School of Medicine, Juntendo University, Tokyo, Japan; c Department of Human Pathology, Graduate School of Medicine, Juntendo University, Tokyo, Japan; d Diagnostics and Therapeutics of Intractable Diseases, Intractable Disease Research Center, Graduate School of Medicine, Juntendo University, Tokyo, Japan

**Keywords:** Encephalomyopathic mitochondrial DNA depletion syndrome, Fatal fulminant pneumonia, F-box and leucine-rich repeat protein 4, Lactic acidosis, Mitophagy

## Abstract

The F-box and leucine-rich repeat protein 4 (*FBXL4*) is a nuclear encoded mitochondrial protein essential for mitochondrial DNA (mtDNA) maintenance. Biallelic variants in *FBXL4* cause FBXL4-related mitochondrial DNA depletion syndrome (FBXL4-MTDPS), characterized by lactic acidosis and developmental delay. We report two siblings diagnosed with FBXL4-MTDPS who died of fulminant pneumonia in infancy; autopsy revealed extensive pulmonary inflammation consistent with severe bacterial infection. FBXL4-MTDPS may involve intrinsic defects in pulmonary infection defense, increasing susceptibility to fatal infection such as pneumonia.

## 1 Introduction

The F-box and leucine-rich repeat protein 4 (*FBXL4*) gene on chromosome 6q16 encodes an F-box protein essential for maintaining mitochondrial DNA (mtDNA) [4]. Biallelic *FBXL4* variants cause FBXL4-related encephalomyopathic mitochondrial DNA depletion syndrome (FBXL4-MTDPS), also known as mtDNA depletion syndrome 13 (OMIM #615471), a rare autosomal recessive disorder with a prevalence of 1 in 100,000–400,000. It typically presents in the neonatal period with lactic acidosis and neurological symptoms [2]. Although early childhood death is common, specific causes are rarely detailed, and genotype–phenotype correlations remain unclear[4]. This study reports the cases of two brothers with a novel *FBXL4* variant who died of fulminant pneumonia. An autopsy was performed on one patient.

## 2 Case presentation

P1 and P2 were the first and third children of healthy, non-consanguineous parents. P1 was delivered vaginally at 38 weeks and 2 days following fetal distress and presented with neonatal asphyxia. He was small for gestational age, with a birth weight of 1934 g (˗2.7 SD), birth length of 45.0 cm (˗1.9 SD), and head circumference of 31.5 cm (˗1.2 SD). Soon after birth, he developed persistent hyperlactatemia and hypoglycemia. At 3 months, growth failure and feeding difficulties necessitated tube feeding. He subsequently experienced recurrent episodes of lactic acidosis, often infection-related. P2 was born at 40 weeks following a prenatal diagnosis of cerebral ventriculomegaly. His birth weight was 2482 g (˗1.3 SD), birth length 47.0 cm (˗1.0 SD), and head circumference 33.0 cm (˗0.3 SD). He developed neonatal lactic acidosis without hypoglycemia and showed persistent growth failure due to poor oral intake.

**Fig. 1:**
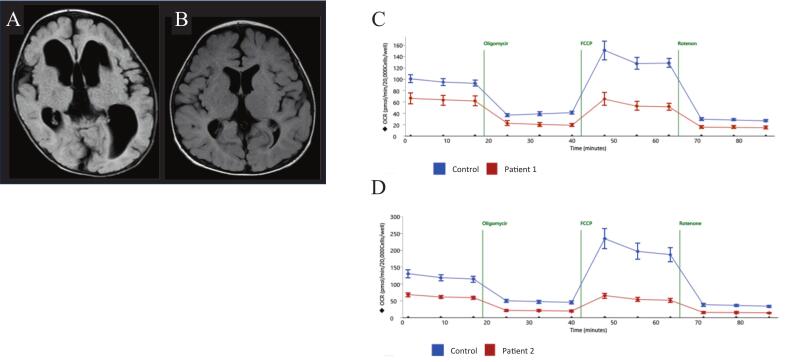
A, B: Fluid-attenuated inversion recovery images from brain magnetic resonance imaging: (A) P1 at 1 year and 9 months, (B) P2 at 1 year and 2 months. Both cases demonstrate reduced cerebral white matter volume, with more pronounced atrophy in P1 than in P2. C, D: Oxygen consumption rate of skin fibroblasts cultured in glucose-containing medium. The blue line represents the control, and red represents the patient. (C) P1 at 5 years and 5 months, (D) P2 at 6 months. Both demonstrated significantly reduced oxygen consumption.

Both were referred to our institution for metabolic and neurological evaluation. At 5 years and 5 months, P1 could sit unsupported but was unable to stand. He had mild hypotonia and dysphagia and exhibited babbling without meaningful speech, with a developmental age of approximately 8 months. At 6 months, P2 lacked head control, could not sit or babble, but demonstrated eye tracking and social smiling, with a developmental age of approximately 3 months. Neither patient had a history of unprovoked seizures nor exhibited clinical signs suggestive of epilepsy. Brain magnetic resonance imaging (P1 and P2) revealed non-progressive white matter volume reduction (Fig. 1A, B) with enlarged ventricles and cerebral cortex atrophy, whereas the brainstem and cerebellum were preserved. P1 also exhibited hypoplasia of the corpus callosum. Magnetic resonance spectroscopy showed lactate peaks in the cerebral white matter, brainstem, and cerebellum in P1, and in the cerebral white matter and basal ganglia in P2. The oxygen consumption rate (OCR) in patient-derived skin fibroblasts, measured using microscale oxygraphy (Seahorse XF96 system; Seahorse Bioscience) as previously reported [15], was reduced in both patients, indicating mitochondrial dysfunction (Fig. 1C, D).

Given suspected mitochondrial disease, mitochondrial cocktail therapy (fursultiamine 10 mg/kg, ascorbic acid 100 mg/kg, biotin 0.5 mg/kg, tocopheryl acetate 10 mg/kg, idebenone 5 mg/kg, and l-carnitine 30 mg/kg) was administered at 5 years 10 months in P1 and 6 months in P2. However, both patients died of fulminant pneumonia. At 6 years, P1 developed cold-like symptoms and experienced cardiac arrest the following morning. Resuscitation failed, and no definitive infectious agent was identified. At 1 year and 10 months, P2 presented with a one-day history of cough and lethargy. Human metapneumovirus was detected, and the patient died within a few hours from severe pneumonia.

**Fig. 2:**
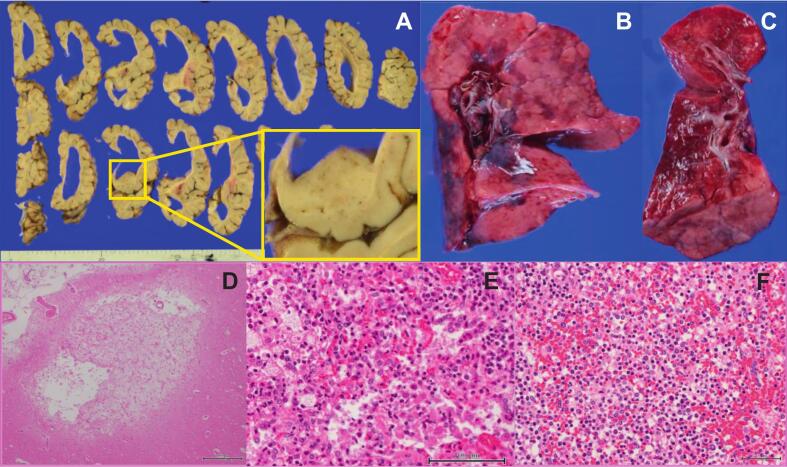
Autopsy findings of P1. A: Histology of the brain shows generalized atrophy of the cerebral cortex and white matter, with ventricular enlargement. The basal ganglia were atrophic, and black discoloration was observed. B, C: Histology of the right lung: (B) the overall view, (C) a sectioned image. Image C demonstrates pleuritis with chest wall whitening, reddish parenchymal discoloration suggestive of congestion, and whitish areas indicating inflammatory infiltration or abscesses. D: Pathological examination of the cerebral white matter at 40× magnification did not reveal a six-layer structure, suggesting neuronal loss. E: Lung pathology at 400× magnification reveals alveolar spaces filled with neutrophil-predominant inflammatory cell infiltration. F: Spleen pathology at 400× magnification shows neutrophilic infiltration in the splenic cords, suggesting a systemic inflammatory response.

An autopsy of P1 revealed normal mitochondrial enzyme activity in the liver, heart, and skin fibroblasts, but non-evaluable low activity in skeletal muscle mtDNA quantification revealed normal levels in the muscle (44.8 %) but reduced levels in the liver (20.8 %, normal >35 %). Histopathology revealed cerebral cortical and white matter atrophy, basal ganglia degeneration, and extensive neutrophilic infiltration in the lungs and spleen, consistent with bacterial pneumonia (Fig. 2). No abnormalities were observed in other organs. Postmortem computed tomography of P2 revealed marked pleural effusion in all lung lobes, suggesting severe pneumonia.

Targeted sequencing of mitochondrial and nuclear genes was performed using genomic DNA from peripheral blood (P1, P2, and parents). Confirmatory analyses were performed using Sanger sequencing. Both siblings harbored compound heterozygous *FBXL4* variants*,* with a maternally inherited frameshift variant in exon 9 (c.1552_1553del; p.Leu518AlafsTer25) and a paternally inherited nonsense variant in exon 5 (c.616C > T; p.Arg206Ter). Both introduce premature stop codons, likely representing loss-of-function variants. According to the ACMG guideline, paternally inherited variant is classified as pathogenic (PVS1, PS1, PM2, PP3), and maternally inherited variants is classified as likely pathogenic (PVS1, PM1, PM2).

Based on clinical presentation, biochemical findings, and genetic results, a diagnosis of FBXL4-MTDPS was established. Clinical and pathological evidence suggested that the siblings died of severe pneumonia.

## 3 Discussion

FBXL4-MTDPS is a cerebral–muscular subtype of mtDNA depletion syndrome, characterized by hypotonia, lactic acidosis, and severe developmental delay[9]. Although some genotype–phenotype associations have been proposed, they remain largely undefined. Biallelic null variants (nonsense, frameshift, splice-site) are generally associated with more severe disease and approximately 30 % survival by age 10, whereas patients with missense variants have milder disease and > 70 % survival beyond 10 years. However, severe neurological symptoms may occur in missense cases, and genotype–prognosis correlations remain uncertain([3,4].

Our cases shared the same compound heterozygous null variants with differing phenotypic severities. P1 was more severely affected. This intrafamilial variability aligns with previous reports[6,7], highlighting that genotype alone does not determine outcome. The maternal variant was a novel frameshift variant with a variant in the leucine-rich repeat (LRR) domain of *FBXL4* (amino acids 340–609), which may mediate protein–protein interactions[11]. Both parental variants likely impair LRR domain function. Approximately 79 % of the missense variants in FBXL4-MTDPS are present within this domain, suggesting that it may be a mutational hotspot[22]. Meanwhile, a milder phenotype has been reported with a variant outside the LRR, supporting a possible domain-specific effect on disease severity[20].

FBXL4 regulates mitochondrial quality by downregulating mitophagy through the ubiquitination and degradation of mitophagy receptors such as NIX and BNIP3 [14]. Loss of this regulation in *Fbxl4* knockout mice leads to excessive mitophagy, resulting in a significant reduction in mitochondrial content and perinatal lethality[1]. Excessive activation or insufficient mitophagy can disrupt mitochondrial function, contributing to disease [16,23]. In severe FBXL4-MTDPS, NIX and BNIP3 accumulation triggers excessive mitophagy and profound mtDNA depletion.

Our patients experienced rapid clinical decline following infection, rapidly progressing to fulminant pneumonia and respiratory failure. However, reports on the clinical course leading to death in patients with FBXL4-MTDPS are limited. A 2017 study documented 11 deaths, with 8 post‑neonatal—5 from sepsis, 2 from pneumonia, and 1 from bleeding. Most reported deaths were preceded by sudden infection-triggered deterioration[7]. To update these findings, we searched PubMed for FBXL4-related articles published between January 2017 and March 2025 and identified five additional post-infancy deaths. Reported causes included sepsis, unspecified viral infections, rhinovirus infections, respiratory infections, and persistent lactic acidosis[10,12,13,17,19]. Overall, 85 % (11/13) of post‑neonatal deaths were infection-related, with sepsis in six cases, underscoring high susceptibility to severe infections. In *Fbxl4* knockout mice, increased expression of mitophagy receptors, specifically in the lung tissue, has been observed[5]. As mitochondria are essential for innate antiviral responses, mitochondrial depletion may impair cellular defense[8]. Of the 11 post‑neonatal deaths, three were directly linked to respiratory infections, and in the six sepsis cases, the infection source was unspecified, and respiratory involvement could not be excluded. Secondary immunodeficiency associated with mitochondrial disorders, including mitochondrial DNA depletion syndromes, has been described in the literature [18,21]. Although immunological evaluations were not available in our patients, such dysfunction may further contribute to their vulnerability to infection.

Conclusively, FBXL4-MTDPS may be involved in intrinsic defects in infection defense, particularly in the lungs. Recognizing this vulnerability is critical for clinical management, including prompt infection control, early intervention for respiratory symptoms, and immune monitoring. Further reports of pathologically and genetically characterized cases are essential to clarify the full spectrum and prognosis of this rare disorder.

## CRediT authorship contribution statement

**Takato Akiba:** Investigation, Writing – review & editing, Writing – original draft. **Shino Shimada:** Supervision, Investigation, Conceptualization, Writing – review & editing, Writing – original draft. **Shimpei Matsuda:** Investigation, Writing – review & editing. **Shoji Ishida:** Investigation, Writing – review & editing. **Yosuke Baba:** Resources, Funding acquisition, Writing – review & editing. **Atsushi Yamashita:** Data curation, Writing – review & editing. **Hiromichi Shoji:** Resources, Writing – review & editing. **Yasushi Okazaki:** Resources, Funding acquisition, Writing – review & editing. **Kei Murayama:** Resources, Investigation, Data curation, Conceptualization, Writing – review & editing.

## Ethical statement

Ethical review was waived because of the nature of the case report, and informed consent was obtained from the parents.

## Declaration of competing interest

There are no commercial or financial relationships that could be perceived as a potential conflict of interest related to this study.

## Data Availability

The datasets generated and analyzed during the current study are available from the corresponding author upon reasonable request.
